# Image Quality Assessment of Digital Radiographs Captured by Hand-Held Devices Versus Wall-Mounted Devices: A Retrospective Comparative Study

**DOI:** 10.7759/cureus.52900

**Published:** 2024-01-25

**Authors:** Turaga Amani, Mouttoukichenin Surenthar, Umamaheswari TN, Roland Prethipa, Lokesh Kumar S

**Affiliations:** 1 Oral Medicine and Radiology, Saveetha Dental College and Hospitals, Saveetha Institute of Medical and Technical Sciences, Saveetha University, Chennai, IND

**Keywords:** wall-mounted, radiography, dental, x-ray unit, hand-held

## Abstract

Background

In diagnostic radiology, the image quality of radiographs is paramount for impeccable diagnosis as it is essential for efficient treatment planning and patient care. In comparison to their well-established wall-mounted equivalents, the growing use of handheld devices raises concerns regarding their diagnostic effectiveness by questioning their image quality. Hence, to fully comprehend the clinical importance of handheld X-ray equipment, it is important to look into their image quality for better diagnostic performance.

Aim

The study aimed to determine the image quality of handheld X-ray units and compare them with wall-mounted X-ray units in routine dental practice based on objectifiable image quality parameters.

Materials and Methods

For the study, 200 digital radiographic images (102 taken using handheld and 98 using wall-mounted X-ray units) were collected randomly from archives, including radiographs with cone-cut and positional errors. Five observers, three faculty members, and two postgraduates, who were all blinded, subjectively judged the image quality using a five-point rating scale for five individual parameters: contrast, sharpness, cone-cut, and error in vertical and horizontal angulations of position indicating device separately. The mean score for all observers was calculated, and statistical analysis was performed using the Mann-Whitney U test. The scoring of one faculty member experienced in oral radiology as baseline data was used to compare interobserver agreement among the other observers.

Results

There is a significant difference between the two groups in cone-cut and error in horizontal angulation. There is no significant difference between the two groups when parameters such as contrast, sharpness, and error in vertical angulation are considered. The images from handheld devices showed better image quality (p = 0.006) compared to the wall-mounted device. There was 87% interobserver agreement between the five observers.

Conclusion

The present study demonstrated a significant difference between the handheld device and the wall-mounted device when all the five parameters including errors are considered to assess the image quality. Hence, handheld devices can be used for regular clinical practice as an alternative to wall-mounted devices. Nevertheless, stringent radiation safety precautions are essential.

## Introduction

In modern clinical dentistry, the utilization of X-ray images significantly influences the quality of diagnosis and treatment. As a result, orthopantomograms and dental X-rays are the preferred methods for radiographic assessments in the common dental practice. Consequently, dental diagnostic imaging stands as one of the most frequently conducted radiological procedures globally. This could potentially affect the overall radiation exposure throughout a person’s lifetime promoting malignancies [1,2]. Portable handheld X-ray machines are often marketed for their ease of use, reduced radiation exposure, and minimal space requirement in the clinic. Evaluation of diagnostic effectiveness includes consideration of the balance of image quality and safety between patients and clinicians. Diagnostic effectiveness paves the way to accurate diagnosis and is essential for efficient treatment planning and patient care in diagnostic radiology. In comparison to their well-established wall-mounted equivalents, the growing use of handheld devices raises concerns regarding their diagnostic effectiveness. Hence, to fully comprehend the clinical importance of handheld X-ray equipment, it is important to look into their image quality for better diagnostic performance [1].

Digital radiography necessitates 90% less radiation compared to E-speed film. Within digital imaging, it is possible to dynamically adjust image attributes post-acquisition for better image resolution, which in turn contributes to providing a better diagnosis. The high resolution of intraoral radiography enhances the visualization of supporting tissues, including periodontal ligament (PDL) space, lamina dura, and bony trabeculae [3,4].

In modern intraoral radiography, portable handheld X-ray devices have emerged as a notable advancement. Unlike the conventional method of using wall-mounted units, handheld devices offer specific advantages, particularly in situations where patients face mobility challenges. For instance, when dealing with non-ambulatory patients, handheld X-ray devices are beneficial due to their mobility and simplicity [5]. They allow for radiographic exams without the need to reposition the patient. This innovation provides flexibility and convenience, especially in cases where traditional wall-mounted units might be less suitable. Forensic dentistry uses radiology to determine the age of an individual by assessing the stage of the eruption of teeth. It is also used to ascertain the evidence in the identification of the suspect and to determine the cause of death [6,7].

Dental radiographs provide essential data that is useful for registering, detecting, gathering, and preserving forensic evidence. Traditionally, handheld X-ray units have established their use in forensics as they prevent superfluous moving and transportation of the remnants and, in some cases, enable a thorough forensic odontology examination right away before the rigor mortis period, excluding body-mutilating treatments [8]. In a previous study by Pittayapat et al., portable handheld X-ray equipment provided adequate image quality for use in forensic odontology [7]. However, the usage of handheld devices in routine dental clinical practice is not much explored in the literature when the pros are upheld.

This portable X-ray device captures radiographs twice as fast as traditional systems, reducing retakes by up to 50%. It is lightweight, cordless, and works with various imaging materials [9]. Its flexibility allows for exposures in different positions, minimizing errors. It is suitable for hospitals, home healthcare, humanitarian efforts, and off-site locations. It is also rechargeable and can operate without a continuous power source [10,11].

To our knowledge, this is the first study to assess the image quality of handheld X-ray units in comparison to conventional wall-mounted units in routine dental setups. This study aimed to determine the image quality of handheld X-ray units and compare them with wall-mounted X-ray units in routine dental practice based on objectifiable image quality parameters. The effectiveness of different observers in assessing the image quality of radiographs was also evaluated from the outcome, having a rationale that image quality of radiographs plays a key role in impeccable diagnostic accuracy and it is crucial when marketing new medical devices as it ensures device safety, efficacy, and compliance with regulatory standards, contributing to patient care and clinical improvement.

## Materials and methods

The Institutional Human Ethical Committee of Saveetha Dental College and Hospitals, Saveetha Institute of Medical and Technical Sciences, Saveetha University, Chennai, India, issued approval IHEC/SDC/OMED-2203/23/210. The current study was carried out on a randomly selected set of 200 intraoral periapical (IOPA) images available from the archives between June 15, 2023, and July 15, 2023, taken by two experienced radio technicians in two different radiology labs: one with wall-mounted and one with handheld X-ray units. The images were taken from patients who had reported to the Department of Oral Medicine and Radiology of our institution for various dental complaints. Having known the fact that the images were regularly stored in unedited form in the archives, they were retrospectively taken for the study.

**Figure 1:**
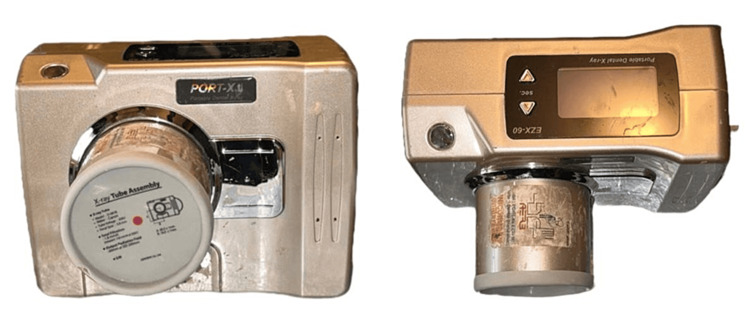
Genoray Port X II handheld dental X-ray unit

In this study, digital intraoral periapical radiographs were used, which were captured using a wall-mounted X-ray device (Confident 70 kvp, 8 mA, 0.4-0.6s, 230 mm focus to the end of the tube) and an FDA-approved handheld X-ray device (Port X II - portable X-ray from Genoray 60 kvp, 2 mA, 0.01-0.04s, 225 mm focus to the end of the tube distance), shown in Figure 1, with a common image receptor - KODAK Carestream RVG 5200 using the bisecting angle technique. Digital intraoral periapical radiographs imaged for various dental pathologies like dental caries, periodontitis, tooth fracture, etc. were included, and it was ensured to include radiographs that exhibited cone cuts, partial images, and positional errors. Bitewing, occlusal, film-based images, and radiographic images, which were edited for appropriate contrast and sharpness were excluded.

The sample size was determined as 200 using G*Power software when an effect size of 0.5, a significance level of 0.05, and a power of 0.80 were set. A set of 200 images (98 images from wall-mounted and 102 images from handheld X-ray units) were collected from archives using the convenience sampling method. They were shuffled and randomized using a simple randomization technique with the help of randomization software (Random Allocation Software v2.0, open-source software). Five observers, three of whom are faculty members and two postgraduates, subjectively judged the image quality using a five-point rating scale between 0 and 5 for five individual parameters namely contrast, sharpness, cone-cut, and errors in vertical and horizontal angulations of position indicating device separately. The observers were blinded to the modality used. A score of 0 indicated very poor image quality, while a score of 5 indicated excellent image quality. Each parameter was individually scored on an Excel spreadsheet, and a total score of 25 was marked to assess the image quality of each radiograph. The assessment was based on clarity, resolution, sharpness, density, and a clear view of the teeth and surrounding structures. Errors in horizontal and vertical angulations and cone cuts were also assessed if they were present in the radiograph. The scoring of one faculty member who is experienced in oral radiology was used as baseline data to compare interobserver agreement with the other four observers.

Statistical analysis

A significant sample size was established using a statistical power analysis (G*Power, free source program). The obtained data from the observers was tabulated and analyzed using IBM SPSS statistics version 23 (IBM Corp., Armonk, NY). The interobserver agreement was analyzed using the kappa statistics. The non-parametric Mann-Whitney U test was performed on the aforementioned parameters, namely contrast, sharpness, cone-cut, and errors in horizontal and vertical angulations, to analyze the image quality. The level of significance was set to p < 0.05. P values below 0.01 were deemed to be extremely significant.

## Results

Table 1 describes that a significant difference (p < 0.05) exists between the two groups, with the handheld X-ray device scoring the highest. This implies that the images obtained from handheld devices are superior to images obtained from the wall-mounted device.

**Table 1:** Comparison of overall image quality between handheld and wall-mounted devices * denotes statistically significant difference.

Type of machine	N (Total number)	Mean	Std. deviation	p-value
Handheld	510	21.40	2.636	0.006*
Wall-mounted	490	20.69	3.399	

Table 2 shows the comparison of image quality concerning the five selected parameters wherein a significant difference was observed between the two groups in cone-cut and error in horizontal angulation. The mean scores were highest for the handheld device in two of these parameters. There is no significant difference between the two groups when parameters such as contrast, sharpness, and error in vertical angulation are considered. However, the mean scores were highest for the handheld device in all of these parameters.

**Table 2:** Comparison of image quality between the handheld and wall-mounted devices with respect to the five parameters * denotes statistically significant difference.

Parameters	Type of machine	Mean	Std. deviation	p-value
Contrast	Handheld (510)	3.71	0.986	0.766
	Wall-mounted (490)	3.66	1.119	
Sharpness	Handheld (510)	3.72	1.046	0.058
	Wall-mounted (490)	3.57	1.141	
Cone-cut	Handheld (510)	4.92	0.307	<0.001*
	Wall-mounted (490)	4.75	0.659	
Error in vertical angulation	Handheld (510)	4.42	0.913	0.575
	Wall-mounted (490)	4.35	1.018	
Error in horizontal angulation	Handheld (510)	4.64	0.692	<0.001*
	Wall-mounted (490)	4.36	0.844	

**Figure 2:**
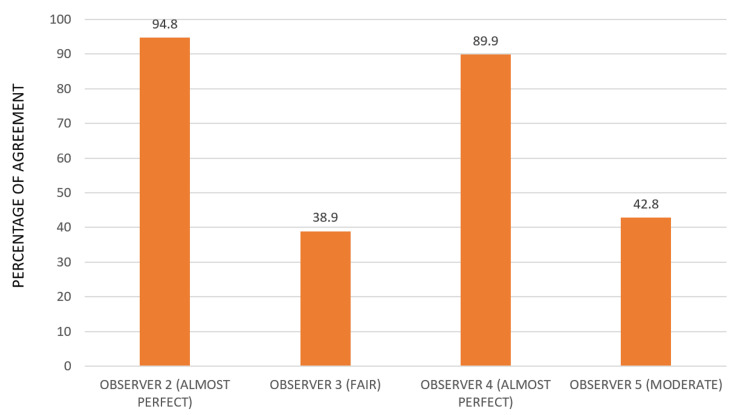
Interobserver agreement using kappa statistics on comparing with the baseline

Figure 2 represents the graphical depiction of interobserver agreement between observers 2, 3, 4, and 5 with observer 1’s scoring taken as the baseline value. Observers 2 and 4 had almost perfect agreement followed by observers 5 and 3 who had moderate and fair agreement, respectively. Certainly, there was no statistical significance among all the four observers when kappa statistics is used.

**Figure 3:**
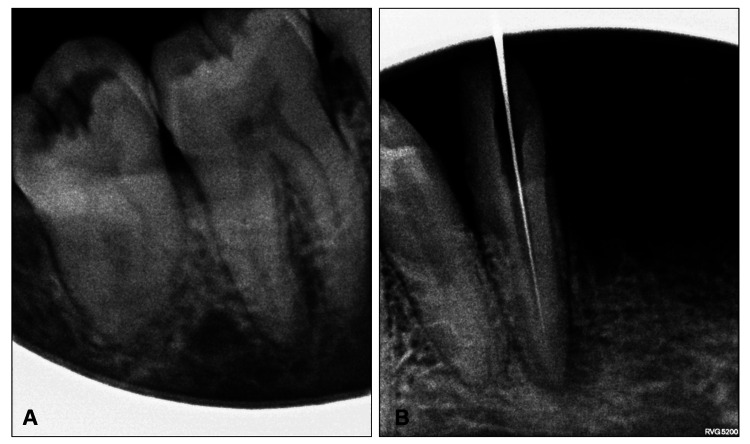
(A and B) Intraoral periapical radiographic images taken using a wall-mounted device

Figure 3 shows intraoral periapical radiographic images taken using a wall-mounted device wherein the image qualities are compromised by the presence of cone-cuts, deranged radiographic contrast, and unsharpness. The represented that sample images were scored least by the observers.

**Figure 4:**
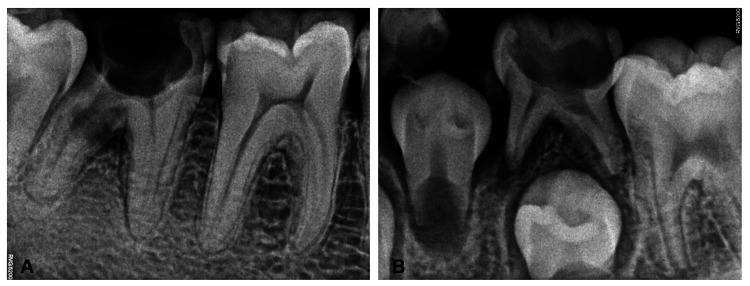
(A and B) Intraoral periapical radiographs taken using a handheld device

Figure 4 shows intraoral periapical radiographic images taken using a handheld device. There is notable good radiographic contrast and sharpness in Figure 4 (Panel A) represented by the delineation of enamel, dentin, and pulp in 46 and recognizable radiolucency involving the furcation area in 47 with sharp demarcation of bone trabeculae. Figure 4 (Panel B) is another example of a better-quality radiograph that displays a wide coverage area covering deciduous and permanent dentition without cone-cut or errors in horizontal and vertical angulations attributed to better positioning of the handheld device. These two sample images received the highest scores from the observers.

## Discussion

The findings of this study indicated there was a noteworthy statistically significant difference in the overall image quality (Table 1), and the handheld device performed better concerning the image quality (Figure 4) compared to the wall-mounted device (Figure 3). It is interesting to note that our findings are consistent with a prior study conducted by Hoogeveen et al. in 2021 [5]. Their study focused on non-inferiority clinical trials involving bitewing images between handheld and wall-mounted devices and revealed that there was no considerable distinction in the preference between the two modalities. The difference in our findings can be attributed to the fact that their study included bitewing radiographs, and they gave scores based on the preference for one of the two or neither based on diagnostic quality. These factors probably played a role in the variation of outcomes. However, the accordance with our study is explained by the fact that although the overall image quality exhibits statistically significant differences, the parameters such as contrast, sharpness, and error in vertical angulation do not show significance (Table 2), implying that both imaging modalities are comparable. Therefore, if a handheld device has better overall diagnostic quality, it should also be similar and comparable to wall-mounted devices so that clinicians can utilize it as an alternative to the latter in routine dental setups.

The same justification also applies to the study carried out by Nitschke et al. in 2020 [1], which also claimed to agree with our findings. Their primary focus revolved around a meticulous evaluation of the image quality derived from two distinct X-ray apparatuses: the HH Nomad Pro 2 and the WM X-ray unit Heliodent Plus. Notably, to ensure accurate comparisons, they maintained uniformity by employing the same digital sensors and consistent examination conditions for both the Nomad Pro 2 and the Heliodent Plus devices. Their investigation delivered a comprehensive comparison of image quality across various radiographic images, encompassing premolars, molars, and bitewing captures. They also found an intriguing alignment of their findings with the research conducted by Ulusu et al. 2014 [12], where it was revealed that handheld devices yielded image quality akin to both digital and conventional bitewing radiographs. This confluence of outcomes underscores the convergence with our study.

In a separate laboratory study conducted by Lommen et al. 2017 [13], both the handheld and wall-mounted devices consistently demonstrated comparable accuracy, resulting in similar image quality. Their investigation involved the examination of bitewing and periapical radiographs, utilizing a tangible tooth phantom. It is noteworthy that our findings are similar to the explanation previously provided above. In their research, Brooks et al. 2009 [14] documented a study where 12 patients needed a full-mouth radiographic series for diagnostic purposes. Half of the radiographs (one side randomly selected) were taken with the handheld Nomad and the other half using wall-mounted dental X-ray equipment. Three reviewers separately scored each image on a three-point scale for diagnostic usefulness and quality. While the structure of their study varied from our retrospective approach, it is important to note that their results were consistent with our findings. Further, their study was similar to our study in the way that it did not use the same radiographic image to compare the imaging modalities.

Previous studies have emphasized that the operator’s training significantly influences the precision of targeting, often surpassing the impact of the device type, whether handheld or wall-mounted. The findings of the study partially corroborate this assertion as there is a similar trend observed. In this study, the percentage of interobserver agreement varied among observers, but no statistically significant differences were found, suggesting plausible agreement. Observer 2 had an agreement of 94.8%, which is considered almost perfect. Observer 3 had an agreement of 38.4%, which is fair. Observer 4 had an agreement of 89.9%, which is also almost perfect. Finally, observer 5 had an agreement of 42.8%, which is moderate. Interestingly, postgraduate students who were observers 4 and 5 performed equally well as faculty members, indicating that they received sufficient training in the field of oral radiology. This is different from the findings of Pittayapat et al.'s study, where the observers had varying dental specializations, resulting in low interobserver agreement [10,15]. In our study, the images were taken by experienced radio technicians, which is not per Hoogeveen et al.'s study where they only used dental students as operators, whereas well-experienced radio technicians were used as operators in this study [16].

The application of the “as low as reasonably achievable (ALARA)” principle may imply a greater emphasis on the handheld device in contrast to a wall-mounted dental X-ray unit. This is because the radiation exposure from the wall-mounted units falls within the range of 0-0.1 mSv, which pertains to a yearly full-body effective dose, whereas the handheld device results in an estimated yearly exposure dose of 0.26 mSv. However, this quantity is still just one-third of the 3.65 mSv background radiation emitted by natural processes annually [17,18]; therefore, it can be a better alternative to wall-mounted devices when the pros are taken into account. According to previous studies to avoid harmful radiation exposure, it is important to follow the Atomic Energy Regulatory Board (AERB) guidelines. X-ray workers should not exceed 20 mSv per year, averaged over five years, while the public should not exceed 1 mSv per year. According to Goren et al.'s study in 2008, it was found that all backscatter measurements from handheld devices were below the maximum radiation limit allowed by the Food and Drug Administration (FDA). Occupational exposure levels were safe, and the film dose was consistent with recommendations [19].

Handheld dental X-ray equipment is lightweight, cordless, and easily transportable. They produce high-quality diagnostic images and require fewer accurate angulations. However, accidents like damage to the equipment, accidental exposure, and failure of the timer can occur. To prevent such accidents, the usage of necessary precautions like thyroid collars and personal dosimetry is recommended [20]. Users of handheld portable X-ray devices must provide proof of radiation safety training to understand risks and protection measures [20-22]. While most of the previous studies conducted the image quality assessment on the same real (in-vivo) or phantom (in-vitro) teeth for both imaging modalities, our study used different teeth randomly collected with the convenience sampling method for each imaging modality. This retrospective structure of the study, in our opinion, would represent the acknowledged study's shortcomings. This was probably the reason for the equivalent results when the same tooth images were compared without a statistically significant difference in all of those studies, whereas our study deviated and showed a substantial difference. Yet another limitation is that the five-point scoring was not standardized under each parameter, and only the observer's expertise and knowledge were used to rate the scores, which could possibly stem intra-observer biases.

The shortcomings of this study necessitate future research to investigate comparative analyses on radiation dose and patient comfort, prospective studies for more reliable data, longitudinal studies for long-term insights, the influence of technical improvements in digital radiography, and studies including a variety of patient demographics.

## Conclusions

The present study demonstrated a significant difference between handheld devices and wall-mounted devices when all the five parameters including errors are considered to assess the image quality. The handheld device showed better image quality when compared to the wall-mounted device. Hence, handheld devices can very well be used for regular clinical practice as an alternative to wall-mounted devices in addition to forensic purposes, provided the radiation safety measures are strictly undertaken. The forthcoming future objectives of our study encompass evaluating the knowledge, attitude, and awareness of private dental practitioners toward the use of portable dental X-ray machines, accomplished through a questionnaire-based survey.
